# Characterization of N-Linked Glycosylation in a Monoclonal Antibody Produced in NS0 Cells Using Capillary Electrophoresis with Laser-Induced Fluorescence Detection

**DOI:** 10.3390/ph6030393

**Published:** 2013-03-21

**Authors:** Melissa Hamm, Yang Wang, Richard R. Rustandi

**Affiliations:** Vaccine Analytical Development, Merck Research Laboratories, West Point, PA 19486, USA; E-Mails: Melissa_hamm@merck.com (M.H.); yang_wang@sinobiological.com (Y.W.)

**Keywords:** CE-LIF, APTS, HILIC-HPLC, glycans, monoclonal antibody NS0, capillary electrophoresis

## Abstract

The N-linked glycosylation in recombinant monoclonal antibodies (mAb) occurs at Asn297 on the Fc region in the CH_2_ domain. Glycosylation heterogeneities have been well documented to affect biological activities such as antibody-dependent cellular cytotoxicity (ADCC) and complement-dependent cytotoxicity (CDC) through their interaction with Fc-receptors. Hence, it is critical to monitor and characterize the N-linked glycosylation profile in a therapeutic protein such as a mAb for product consistency. In one approach, the glycans are first released from the mAb using an enzyme specific digestion, such as Protein N-Glycosidase F (PNGase) and subsequently they are labeled using a fluorophore, for example, 8-aminopyrene-1,3,6-trisulfonic acid (APTS) . Here we have applied this approach and used Capillary Electrophoresis with Laser-Induced Fluorescence detection (CE-LIF) to analyze a recombinant mAb produced in murine myeloma (NS0) cells. The technique provides short analysis times, efficient separations, and high sensitivity. CE-LIF peak identification was done by a combination of glycan standards and treatment with various exoglycosidases. Furthermore, the APTS-labeled glycans were also analyzed using hydrophilic interaction chromatography (HILIC) high performance liquid chromatography (HPLC) to aid identification of minor peaks by sample collection and off-line mass spectrometry (MS) analysis.

## 1. Introduction

Development of monoclonal antibodies (mAb) as therapeutics in the biopharmaceutical industry, including biosimilar or biobetter versions of marketed mAbs has increased tremendously over the last ten years [1,2,3]. A recombinant mAb is a glycoprotein which contains a conserved N-linked glycosylation site at Asn297 on the Fc region in the CH_2_ domain [4]. There are many known physical functions of N-linked glycosylation in a mAb such as affecting its solubility and stability [5,6,7], protease resistance, binding to Fc receptors, cellular transport and circulatory half-life *in vivo* [8,9,10,11]. In addition, there are also known biological functions of N-linked glycosylation in a mAb which are related to the micro-heterogeneities of glycan structures. For example, the absence of a core fucose residue [12] and the presence of a bisecting N-acetylglucosamine (GlcNAc) enhance the ADCC activity [13]. A decrease in sialic acid containing glycans may also play role in elevating ADCC activity [14]. Finally, terminal galactose residues in biantennary glycans may affect the CDC activity [15]. 

The micro-heterogeneities of mAb glycosylation depend on the expression system as well as clone and various growth conditions such as cell culture media, temperature and time. Therefore, it is very important to have analytical tools that can quantitate and monitor the glycosylation pattern. There are many analytical methods that are commonly used to analyze glycosylation such as NMR, MS, HPLC and CE. The most commonly used quantitative tools to analyze glycosylation are HPLC either with pulsed amperometric detection (PAD) [16,17] or with fluorescence detection employing fluorescently-labeled glycans [18,19,20] and CE with a LIF detector for various fluorescently-labeled glycans [21,22,23]. CE-LIF technique with APTS-labeled glycans is routinely employed in biopharmaceutical industries to analyze the glycosylation heterogeneities in a mAb. This is because the three negatively charged sulfonic groups in APTS attached to the glycans provide a high efficiency separation, fast analysis time, and high sensitivity detection to low attomole range [24,25,26].

A peak characterization strategy for APTS labeled glycans most commonly uses a combination of glycan standards and exoglycosidase-treatments. In addition, CE-LIF coupling with MS analysis has also been demonstrated by several groups [27,28,29] and most recently Gahoual *et al.* [30] describe the first characterization of trastuzumab with 100% sequence coverage including main glycoforms using sheathless CE-MS, however, this technology is still difficult to implement in a normal laboratory setting for routine testing. Hence, characterization of minor peaks in CE-LIF remains a challenging process. Here, we report characterization of N-linked glycosylation in a mAb produced in NS0 cells using a combination of CE-LIF and HILIC HPLC of APTS-labeled glycans including off-line MS analysis for confirmation.

## 2. Experimental Section

### 2.1. Reagents

All reagents were analytical grade unless otherwise noted. Phosphate buffer saline (PBS) was obtained from an internal Merck buffer service. Carbohydrate separation buffer and APTS dye solvent were obtained from Beckman Coulter (Fullerton, CA, USA). High purity APTS was purchased from either Fluka (Milwaukee, WI, USA) or Invitrogen (GE Healthcare, Uppsala, Sweden). Sodium cyanoborohydride (NaBH_3_CN), β-mercaptoethanol (βME), acetic acid (CH_3_COOH), ε-aminocaproic acid (EACA), hydroxypropylmethylcellulose (HPMC), ammonium acetate (CH_3_COONH_4_), acetonitrile (CH_3_CN), 200 absolute proof ethanol, as well as various exoglycosidase enzymes, β-galactosidase, β-N-acetylglucosaminidase, α-mannosidase, α-fucosidase and glycan standards, G0-GlcNAc, G0F, G0, G2F, G2, Man5, A2F, A2, A1F, A1 were purchased from Sigma Aldrich (St. Louis, MO, USA). CE-grade water was purchased from Microsolv (Eatontown, NJ, USA). Nonidet NP-40 detergent, SDS 10% (w/v) solution and protein desalting columns were purchased from Thermo-Fisher (Waltham, MA, USA). The PNGase enzyme was purchased from New England Biolabs (Ipswich, MA, USA). The Sialidase A (α-neuraminidase) enzyme and its reaction buffer were purchased from Prozyme (Hayward, CA, USA).

### 2.2. Preparation of mAb Samples

All monoclonal antibodies were produced in mice myeloma NS0 cells and were purified to ≥99% by the Bioprocess Research and Development group at Merck Research Laboratories (Merck & Co. Inc., West Point, PA, USA). Their sample concentrations were measured using UV/Vis spectrophotometry with known extinction coefficients.

### 2.3. PNGase Digestion to Remove Glycans from mAb

Approximately 300 µg protein is dried and resuspended in 45 µL PBS, 1.5 µL 5% SDS, and 1 µL β-mercaptoethanol (1:10 diluted in water). This mixture is heated at 37 °C for 10 minutes to denature the mAb, then 5 µL NP-40 and 10 µL PNGase (10,000 unit/mL) are added followed by an overnight incubation at 37 °C. Three volumes of cold ethanol were added to precipitate the protein and the supernatant containing glycans is subsequently removed and dried using a SpeedVac (Thermo Scientific, Waltham, MA, USA).

### 2.4. APTS Labeling and Excess Dye Removal

The dried, isolated glycans are incubated in the presence of 2 μL of sodium cyanoborohydride and 2 μL of 50 mg/mL APTS at 60 °C for 2 hours. The reaction is stopped with the addition of 46 μL of CE-grade water. The labeled glycans can be further diluted in water for CE-LIF analysis. For HPLC analysis, the excess APTS dye needs to be removed and the removal is performed using G10 MiniTrap cartridges (GE Healthcare, Uppsala, Sweden). The cartridges are used according to the manufacturer’s recommendation and are equilibrated in 10 mM acetic acid. A maximum sample volume of 300 µL is loaded onto each column and allowed to completely enter the column bed. A stacker volume of 700 µL of 10 mM acetic acid is added to the column and allowed to completely enter the column bed. A volume of 500 µL of 10 mM acetic acid is added and the flow through is discarded. Another volume of 500 µL of 10 mM acetic acid is added and the flow through is collected and dried using a SpeedVac. The dried sample is resuspended in 1 mL CE-grade water. The efficiency of excess dye removal is assessed using CE prior to HPLC analysis.

### 2.5. Exoglycosidase-Treatments

Following PNGase-treatment and APTS-labeling, the glycans were treated with β-galactosidase, β-*N*-acetylglucosaminidase, α-mannosidase, and α-fucosidase in sequence using the reaction buffers provided by the manufacturer. A portion of the sample post individual enzyme treatment was kept for analysis. All enzymatic digestions were incubated at 37 °C for 16–20 hours. The α-neuraminidase enzyme-treatment was performed in the same manners as all of the other enzymes in the glycan mixture.

### 2.6. CE-LIF Separation

A detailed description of this method has been published previously [31]. Briefly, separations were performed on a Beckman Coulter PA800 CE system equipped with LIF detector using an Argon ion laser (Fullerton, CA, USA); λ_ex_ = 488 nm, λ_em_ = 520 nm. Polyvinyl Alcohol-Coated (PVA) capillaries of 50 µm ID and 365 µm OD with L_T_ = 50 cm and L_D_ = 40 cm were obtained from either Beckman Coulter (Fullerton, CA, USA) or Agilent (Santa Clara, CA, USA). The capillary was equilibrated with carbohydrate buffer from Beckman Coulter for 2 minutes at 30 psi. Separation was conducted using the carbohydrate separation buffer from Beckman Coulter at 30 kV (reverse polarity) for 20 min. Samples were injected neat for 20 sec at 0.5 psi except for samples that were obtained from the HPLC fraction collection which were injected for 40 sec at 0.5 psi. The current generated during separation is around −15 ± 2 µA. Separation for G0 and Man5 was conducted using a new buffer (40 mM EACA/CH_3_COOH in 0.2% HPMC). The rest of CE parameters are similar as above for Beckman buffer, except the current generated during separation is −11 ± 2µA.

### 2.7. HPLC Separation

The APTS-labeled glycans were separated using a TSKgel Amide-80 HPLC column (25 cm × 4.6 mm ID, 5 µm particle size, 80 Å pore size) (Tosoh Bioscience LLC, King of Prussia, PA, USA) employing a Waters Alliance Model 2695 liquid chromatography system equipped with a fluorescence detector (Model 2475, Milford, MA, USA). The column temperature was kept at 25 °C and the excitation wavelength was 425 nm and the emission wavelength was 505 nm. The flow rate was 1 mL/min. Separation was performed using a linear gradient of 80% acetonitrile/20% 20 mM ammonium acetate to 40% acetonitrile/60% 20 mM ammonium acetate over 2 hours. The column was equilibrated with 95% acetonitrile for 10 min and a 20 minute equilibration using the gradient starting condition. Peaks between 44–61 minutes were collected over 7 days and injected on CE and HPLC for confirmation. 

## 3. Results and Discussion

### 3.1. N-Linked Glycosylation Profile for mAb Produced in NS0

**Figure 1:**
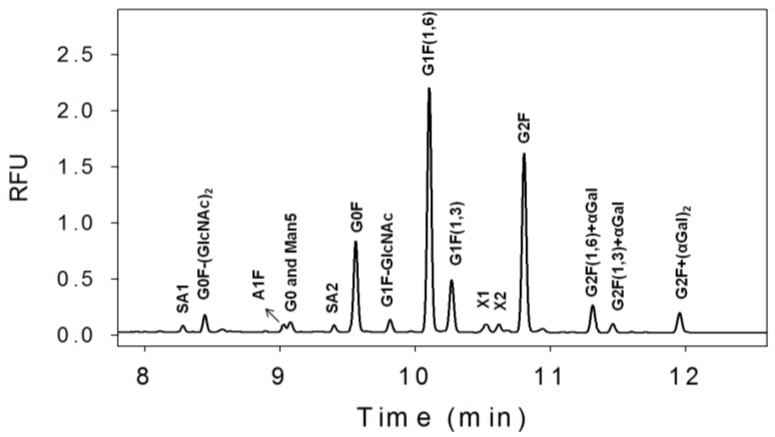
The N-linked glycan profile from a mAb produced in NS0 cell line evaluated using CE-LIF. A nearly complete peak assignment was achieved except for X1, X2, SA1, and SA2. SA = sialic acid containing glycan. See Figure 4A for tentative X1 and X2 assignments.

**Figure 2:**
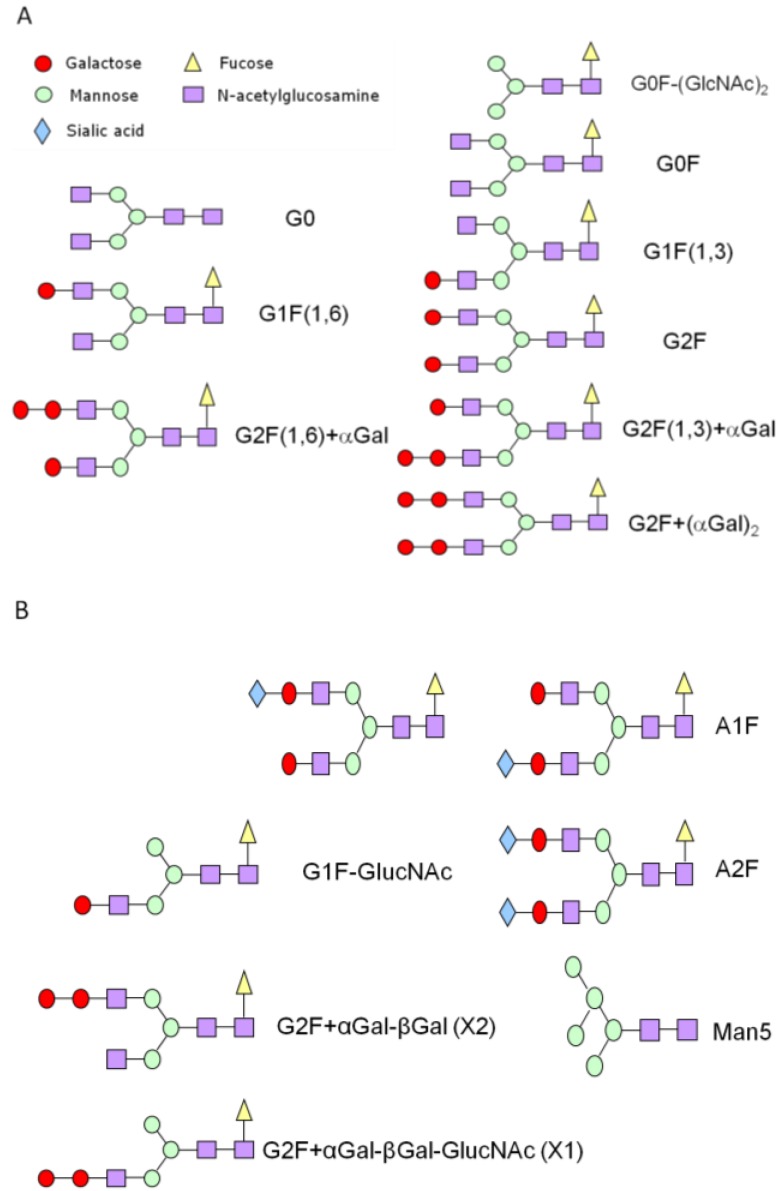
Various biantennary oligosaccharide (glycan) structures relevant to this study.

The N-linked glycans from a mAb produced in NS0 were first released from the protein using a specific enzyme, PNGase, which removed the glycans from the Asn297 position in the Fc region of the mAb. The optimized removal condition was monitored using a CE-SDS gel method previously described [32]. The APTS-labeled glycans were separated using CE-LIF and the electropherogram profile is shown in Figure 1. There are four major peaks observed in the electropherogram, G0F, G1F(1,6), G1F(1,3), and G2F which are typical for a mAb produced in either Chinese Hamster Ovary (CHO) or NS0 cell lines (see Figure 2 for glycan structures). In addition to those four major peaks, there are other smaller glycan peaks observed in this mAb produced in NS0. Some of these minor peaks are not normally observed for a mAb produced in CHO such as G2F+αGal and G2F+(αGal)_2_ [33,34]. 

There are three different sialic acid (SA) containing glycans observed in this mAb indicated by SA1, SA2, and A1F. The exact identity of SA1 and SA2 are still unknown. It is well established that a mAb produced in NS0 could have a small amount of *N*-glycolylneuraminic acid (Neu5Gc) containing glycans which are known to be immugenic in humans [35]. The presence of Neu5Gc in SA1 and SA2 cannot be confirmed at this time. However, the amount of the three combined sialic acid species range from 1%–3% for various clones and clone 3, the clone selected for further mAb development contains the least amount of sialic acid (see Table 1). 

**Table 1:** % Glycan compositions from three different clones.

% Glycan	Clone 1	Clone 2	Clone 3
Sialic Acids	3	1	1
G0F-(GlcNAc)_2_	4	3	2
G0 and man5	3	2	8
G0F	16	33	37
G1F-GlcNAc	2	1	9
G1F	43	44	35
G2F	24	14	6
G2F+αGal	4	2	1
G2F+(αGal)_2_	2	1	1

G1F is the sum of both G1F(1,6) and G1F(1,3); similarly G2F+αGal is the sum of both G2F(1,6)+αGal and G2F(1,3)+αGal.

Glycans with an additional α-1,3 galactose, G2F(1,6) + αGal, G2F(1,3) + αGal, and G2F + (αGal)_2,_ were also observed ranging from 1%–4% (Table 1). These additional α-1,3 galactose glycans may pose a clinical concern since they are also known to give an immunogenic response in humans [33,36,37,38]. However, their relative amount is small plus there are several mAbs on the market that are produced in NS0 and these have no adverse events reported that are linked to these α-1,3 galactose glycans. Several other glycans G0, Man5, G1-GlcNAc, X1, X2 were also observed in varying amounts. Three glycans, A1F, G0, and Man5 co-migrate with this separation condition, hence a different separation buffer was developed to improve the resolution (see Section 3.4). The peak identification strategy is described in Section 3.2 and Section 3.3 including the tentative assignment of X1 and X2.

Part of the clone selection criteria is to either increase or reduce the specific glycan forms. For example, a mAb produced for oncology is desired to have less core fucose and sialic acid residues to increase the ADCC activity. In many cases, Man5 glycans are undesirable considering they have been reported to contribute to higher clearance rates from serum [39]. This was partly achieved for Clone 3 as compared to Clones 1 and 2 indicating that G0 and Man5 have increased while sialic acids decreased. While for another biological activity, CDC, it is expected to be affected by reducing the amount terminal β-galactose of G1F and G2F in Clone 3 [15,40]. Finally, the amount of G2F+αGal and G2F+(αGal)_2_ glycans are also reduced in Clone 3 to avoid any possible immune response.

### 3.2. Exoglycosidase

The commercially available glycan standards were first used to identify the peaks. These glycans G0F, G2F, Man5, A2F, A1F, G0F-(GlcNAc)_2_ were obtained from Sigma-Aldrich. The non-fucosylated glycan standards, G0, G1(1,6), G1(1,3), G2, A1, and A2 were either obtained in-house or purchased from Sigma-Aldrich. They were labeled with APTS using the same procedure as in the glycan sample mixture from mAb treatment and run individually to match the peaks (data not shown).

**Figure 3:**
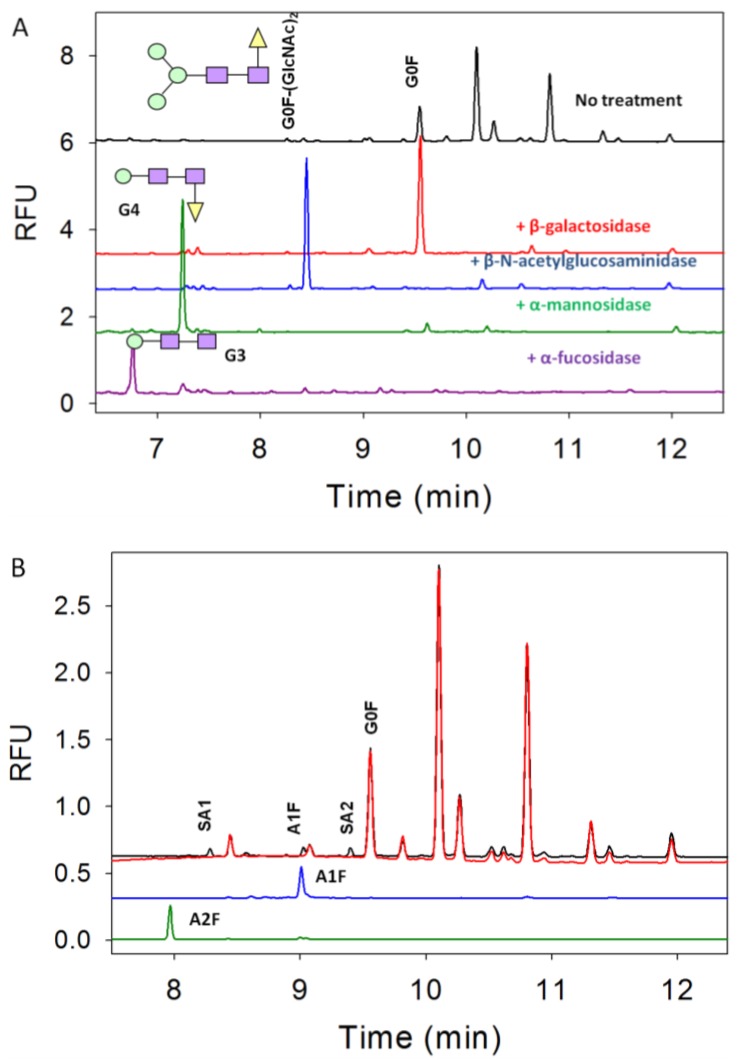
Exoglycosidase treatment of the *N*-glycan mixture. (**A**) Sequential treatment first with β-galactosidase (red trace), second with β-*N*-acetylglucosaminidase (blue trace), third with α-mannosidase (green trace), and finally with α-fucosidase (purple trace); (**B**) *N*-glycan mixture treated directly with α-neurominidase to remove sialic acid attached to the glycans. The non-treated sample (black trace) and treated sample (red trace) were overlaid illustrating that three peaks labeled as SA1, SA2, and A1F disappear upon treatment. A1F and A2F standards were run to compare and confirm the A1F assignment. The assignment of SA1 and SA2 are still not known.

The sequential exoglycosidase experiments were demonstrated in Figure 3A. First, β-galactosidase was added to the glycan mixture and the G1F(1,6), G1F(1,3), G2F, G2F(1,6)+αGal, and G2F(1,3)+αGal disappeared, while G2F+(αGal)_2_ stayed since this glycan needs a specific α-galactosidase enzyme to be altered. Although the signal intensity for G2F+(αGal)_2_ decreases after treatment with β-galactosidase, this could be attributed to possible α-galactosidase impurities present in the β-galactosidase reagent or a much slower cleavage reaction by the β-galactosidase due to a lack of specificity. As a result of this treatment, an expected strong G0F peak appears (red trace). Then this β-galactosidase treated sample was incubated with the β-N-acetylglucosaminidase enzyme to remove the two GlcNAc residues in G0F yielding G0F-(GlcNAc)_2_ (blue trace). Subsequently this sample was treated with α-mannosidase to remove the two mannose residues yielding glycan with only four residues, G4 (Fucose-GlcNAc-GlcNac-Man) (green trace). Finally, this sample was treated with α-fucosidase to yield glycan with only three residues, G3 (GlcNAc-GlcNAc-Man) (purple trace). The migration times of these three and four glycan residues match well with glucose standard units (data not shown). Sequential treatment and a comparison with the glycan standards have confirmed the G2F+(αGal)_2_, G2F(1,6)+αGal, G2F(1,3)+αGal, G2F, G1F(1,6), G1F(1,3), G0F, and G0F-(GlcNAc)_2_ peaks. Furthermore, separate non-labeled released glycans were also analyzed with matrix assisted laser desorption ionization (MALDI) time-of flight (TOF) MS which confirmed the existence of those glycans (data not shown).

The confirmation of sialic acid-containing glycans was determined using a α-neuraminidase enzyme as shown in Figure 3B. The glycan mixture was treated with α-neuraminidase and as a result there are three peaks that disappeared indicated as SA1, A1F, and SA2 (red trace). The identity of A1F was further confirmed using a glycan standard (blue trace). Neither SA1 nor SA2 correspond to A2F, A2 or A1 (A1 and A2 standards not shown). Although the sialic acid-containing glycans have similar size as G2F+αGal, they are expected to migrate faster than other glycans since they possess an additional negative charge. The two sialic acid containing glycans (A2F, A2) migrate earlier than mono sialic acid-containing glycan (A1F, A1). The absolute assignments of SA1 and SA2 cannot be determined at this time even with the use of MALDI TOF MS due to their low abundance.

### 3.3. Characterization of G1F-GlcNAc Using HPLC

During clone selection for mAb development the peak labeled as G1F-GlcNAc has increased dramatically (see Clone 3 in Table 1), while immunogenic glycans, G2F(1,6)+αGal, G2F(1,3)+αGal, and G2F+(αGal)_2_, have decreased. Early on in development the amount of this G1F-GlcNAc peak was 1%–2% hence identification was not needed, however once the amount has increased dramatically, it becomes necessary to positively identify its structure. In order to confirm the G1F-GlcNAc peak, enzymatic digestion along with HPLC fraction collection and subsequent mass spectrometry analysis was employed.

**Figure 4:**
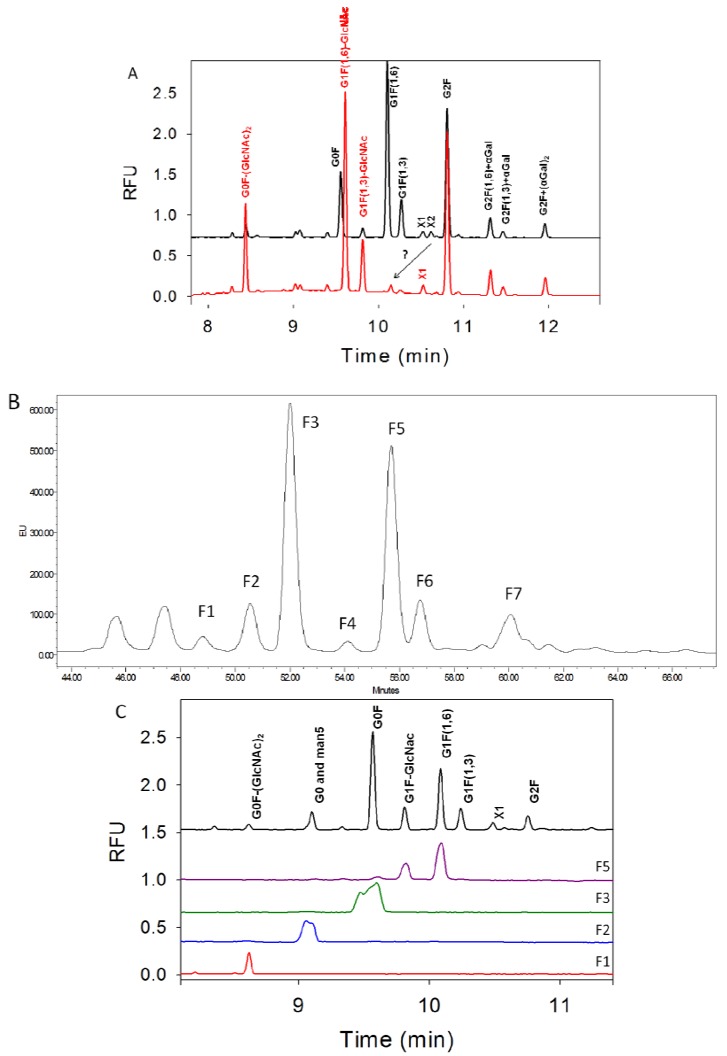
G1F-GlcNAc peak assignment. (**A**) The glycan mixture was treated directly with β-*N*-acetylglucosaminidase (red trace). Upon treatment, removal of the GlcNAc residue causes the G1F(1,3) peak to shift to the same position as the expected G1F-GlcNac. The arrow with a question mark indicates the possible shift of X2 peak. (**B**) The HPLC chromatogram of the separated APTS-labeled glycans. Fractions collected are indicated as F1 through F7. (**C**) All collected fractions from HPLC were reinjected onto CE and Fraction 5 contains the expected G1F-GlcNAc and G1F(1,6) peaks. MS analysis confirms the identity of these peaks. Note that the CE peak shapes for F1 and F2 were distorted because the injection was overloaded but they clearly contain the G0 and man5 peaks for F2 and G0F for F3. HPLC Fraction 4 gives a very low signal on the CE (electropherogram not shown) and could not be determined by MS. HPLC Fraction F5 and F6 were reinjected onto CE and they correspond to G1F(1,3) and G2F, respectively, as expected (CE electropherograms not shown).

Figure 4A shows the use of GlcNAc specific enzyme β-N-acetylglucosaminidase to remove any GlcNAc residues from the glycan mixture sample. Upon treatment the G0F, G1F(1,6), and G1F(1,3) peaks have shifted while G2F(1,6)+αGal, G2F(1,3)+αGal, and G2F+(αGal)_2_ peaks did not change. The treated G1F(1,3) becomes G1F(1,3)-GlcNAc or simply called G1F-GlcNAc and its peak co-migrates with the peak labeled as G1F-GlcNAc (~9.85 min) in the original sample. This provides a good indication that peak at about 9.85 min is indeed G1F-GlcNAc. Further analysis of other glycan peaks indicate that the X1 peak does not move while the X2 disappears and potentially shifts (see arrow) although it is difficult to confirm this shift since the signal intensity is relatively small. Because the X1 peak does not shift, this glycan therefore contains no terminal GlcNAc residue and is tentatively assigned as G2F+αGal-βGal-GlcNAc (see Figure 2B). Peak X2 has disappeared indicating that this glycan peak contains a GlcNAc residue and it migrates after X1 in the electropherogram of the original sample indicating it is larger than X1, therefore this is tentatively assigned as G2F+αGal-βGal (see structures in Figure 2B). Furthermore, the tentative glycan structure assignments for X1 and X2 have corresponding glucose units of 9 and 10 which are similar in migration time to G1F and G2F, respectively.

In order to further confirm the existence of G1F-GlcNAc, an HPLC-based separation was developed with the purpose of collecting the separated fractions for identification on CE and MS. A TSKgel Amide-80 column was used to separate these APTS-labeled glycans. In order to perform an HPLC separation, it was necessary to remove the excess APTS dye using a G10 gel filtration column. The APTS dye removal efficiency was evaluated using CE since all free APTS dye migrates early, between 4–7 min. The best separation was achieved using a long two hour gradient and is shown in Figure 4B. Although at first glance the peak profile is similar between HPLC and CE, later it was discovered that the HPLC separation was not as efficient as compared to CE (see Figure 4C), but it was good enough for MS characterization and confirmation of glycan peaks. Collection of the indicated fractions in HPLC were performed over a one week period and pooled fractions were concentrated and injected on the CE for confirmation (see Figure 4C) and for MS identification. Note that the first two peaks in HPLC at 45.5 and 47.2 minutes before F1 were also collected but there was no detectable signal on CE injection indicating that those peaks could be free dye-related. CE analysis of the HPLC fractions demonstrate that the putative G1-GlcNAc peak was not separated as well on the HPLC as it appears as 2–3 peaks in CE (Fraction 5). But when this particular Fraction 5 was analyzed with MS, the results have confirmed that this fraction contains a small amount G0F, G1F-GlcNAc, and G1F (MS cannot differentiate between G1F(1,6) and G1F(1,3) since both have same mass). Another confirmation of G1F-GlcNAc peak is from the analysis of the non-labeled released glycans using MALDI-TOF MS. All MS data are not shown since they will be the subject of a separate publication.

### 3.4. Separation of Co-Migrating Peaks of A1F, G0, and Man5

The use of a commercially available CE-separation buffer (Beckman buffer) is good enough to separate most of the important glycans. However, three glycans, A1F, G0, and Man5 co-migrate which hinders their quantitation in the sample. The quantitation of sialic acid and A1F can be done by comparing the peak before and after α-neuraminidase treatment. In the final selected clone, there is no detectable A1F species hence the separation method only needs to be developed between G0 and Man5.

**Figure 5:**
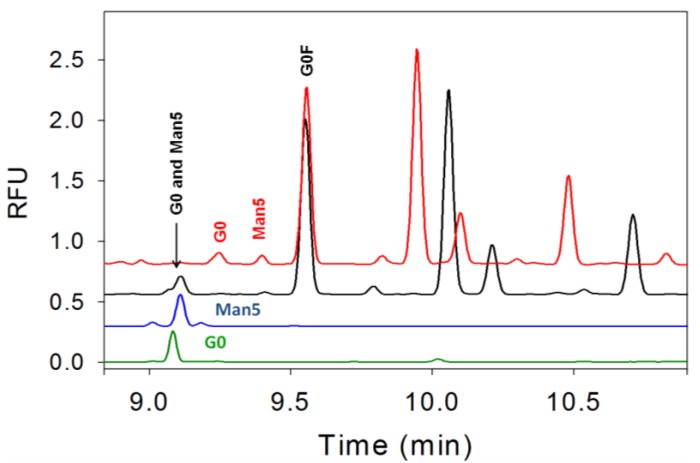
Two glycans, G0 and Man5, co-migrate in the Beckman buffer (black trace). G0 (green trace) and Man5 (blue trace) standards were run separately using the Beckman buffer to confirm the co-migration. An improved separation buffer containing EACA/CH_3_COOH with HPMC was developed and experiments with this buffer show that it was capable of separating G0 and Man5 (red trace).

The separation buffer was optimized using combination of EACA/CH_3_COOH at different concentrations (20–100 mM EACA) and in the absence or presence of HPMC (0.1%–0.5%). The final method that provides good separation is 40 mM EACA/CH_3_COOH with 0.2% HPMC. The electropherograms using this buffer are shown in Figure 5 where G0 and Man5 are well-separated in 40 mM EACA/CH_3_COOH with 0.2% HPMC buffer (red trace) compared to the Beckman buffer (black trace). The G0 and Man5 standards were run using the Beckman buffer to illustrate the co-migration. This new separation method was subsequently employed to evaluate and quantitate the G0 and Man5 content in the sample.

## 4. Conclusions

In this study, we have identified all the major and most of the minor glycans in a mAb produced in NS0 using a CE-LIF method. We have assigned almost all peaks observed in CE-LIF except few minor peaks (1%–2%) using combination of glycan standards, exoglycosidase treatments, HPLC, and off-line MS. The HILIC HPLC method used to separate APTS-labeled glycans is useful for final identification in conjunction with MS. To our knowledge, this is the first APTS-labeled glycan analysis using the combination of CE, HPLC, and MS techniques. We have also developed a separation buffer for use on CE-LIF to separate G0 and Man5 peaks using a combination of EACA/CH_3_COOH and HPMC solutions. The CE-LIF technique has proven to be an indispensable tool for monitoring N-linked glycans in therapeutic mAb development because it is robust, offers high resolution and is quick. N-linked glycan analyses using CE-LIF have been established in quality control environments for glycan profile, identification, product release, clone selection, and is a powerful characterization tool for product consistency. As a consequence, N-linked glycan analysis using CE-LIF provides both great analytical capacity (high information content in a relatively short time) and product coverage for a wide spectrum of mAb products in development (discovery, preclinical, clinical, and commercialization) in the biopharmaceutical industry.

## References

[1] Berkowitz S.A., Engen J.R., Mazzeo J.R., Jones G.B. (2012). Analytical tools for characterizing biopharmaceuticals and the implications for biosimilars. Nat. Rev. Drug Discov..

[2] Beck A., Sanglier-Cianferani S., Van Dorsselaer A. (2012). Biosimilar, Biobetter, and Next Generation Antibody Characterization by Mass Spectrometry. Anal. Chem..

[3] Beck A., Wagner-Rousset E., Ayoub D., van Dorsselaer A., Sanglier-Cianferani S. (2013). Characterization of Therapeutic Antibodies and Related Products. Anal. Chem..

[4] Sutton B.J., Phillips D.C. (1983). The three-dimensional structure of the carbohydrate within the Fc fragment of immunoglobulin G. Biochem. Soc. Trans..

[5] Ghirlando R., Lund J., Goodall M., Jefferis R. (1999). Glycosylation of human IgG-Fc:influences on structure revealed by differential scanning micro-calorimetry. Immunol. Lett..

[6] Liu H., Bulseco G.G., Sun J. (2006). Effect of posttranslational modifications on the thermal stability of a recombinant monoclonal antibody. Immunol. Lett..

[7] Wu S., Luo J., O’Neil K., Kang J., Lacy E.R., Canziani G., Baker A., Huang M., Tang Q., Raju T.S. (2010). Structure-based engineering of a monoclonal antibody for improved solubility. Protein Eng. Des. Sel..

[8] Raju T.S., Scallon B. (2007). Fc glycan terminated with *N*-acetylglucosamine residues increase antibody resistance to papain. Biotechnol. Prog..

[9] Okazaki A., Shoji-Hosaka E., Nakamura K., Wakitani M., Uchida K., Kakita S., Tsumoto K., Kumagai I., Shitara K. (2004). Fucose depletion from human IgG1 oligsaccharide enhances binding enthalpy and association rate between IgG1 and FcγRIIIa. J. Mol. Biol..

[10] Gala F.A., Morrison S.L. (2002). The role of constant region carbohydrate in the assembly and secretion of human IgD and IgA1. J. Biol. Chem..

[11] Wright A., Sato Y., Okada T., Chang K., Endo T., Morrison S. (2000). In vivo trafficking and catabolism of IgG1 antibodies with Fc associated carbohydrates of differing structure. Glycobiology.

[12] Kanda Y., Yamane-Ohnuki N., Sakai N., Yamano K., Nakano R., Inoue M., Misaka H., Iida S., Wakitani M., Konno Y. (2006). Comparison of cell lines for stable production of fucose-negative antibodies with enhanced ADCC. Biotechnol. Bioeng..

[13] Ferrara C., Brunker P., Suter T., Moser S., Puntener U., Umana P. (2006). Modulation of therapeutic antibody effector functions by glycosylation engineering: Influence of Golgi enzyme localization domain and co-expression of heterologous β 1,4-N-acetylglucosaminyltransferase III and Golgi α-mannosidase II. Biotechnol. Bioeng..

[14] Scallon B.J., Tam S.H., McCarthy S.G., Cai A.N., Raju T.S. (2007). Higher levels of sialylated Fc glycans in immunoglobulin G molecules can adversely impact functionality. Mol. Immunol..

[15] Hodoniczky J., Zheng Y.Z., James D.C. (2005). Control of recombinant monoclonal antibody effector functions by Fc N-glycan remodeling *in vitro*. Biotechnol. Prog..

[16] Hardy M.R., Townsend R.R., Lee Y.C. (1989). Separation of Oligosaccharides using High Performance Anion-Exchange Chromatography with Pulsed Amperometric Detection. Methods Enzymol..

[17] Spellman M. (1990). Carbohydrate Characterization of Recombinant Glycoproteins of Pharmaceutical Interest. Anal. Chem..

[18] Guile G.R., Rudd P.M., Wing D.R., Prime S.B., Dwek R.A. (1996). A rapid high-resolution HPLC method for separating glycan mixtures and analyzing oligosaccharide profiles. Anal. Biochem..

[19] Anumula K.R., Dhume S.T. (1998). High resolution and high sensitivity methods for oligosaccharide mapping and characterization by normal phase HPLC following derivatization with highly fluorescent anthranilic acid. Glycobiology.

[20] Kamoda S., Nomura C., Kinoshita M., Nishiura S., Ishikawa R., Kakehi K., Kawasaki N., Hayakawa T. (2004). Profiling analysis of Oligosaccharides in antibody pharmaceuticals by capillary electrophoresis. J. Chromatogr. A.

[21] Klockow A., Amado R., Widmer H.M., Paulus A. (1995). Separation of 8-aminonaphthalene-1,3,6-trisulfonic acid-labelled neutral and sialylated N-linked complex oligsaccharides by capillary electrophoresis. J. Chromatogr. A.

[22] Evangelista R.A., Liu M.-S., Chen F.-T.A. (1995). Characterization of 9-Aminopyrene-1,4,6-trisulfonate-Derivatized Sugars by Capillary Electrophoresis with Laser-Induced Fluorescence Detection. Anal. Chem..

[23] Kamoda S., Ishikawa R., Kakehi K. (2006). Capillary electrophoresis with laser-induced fluorescence detection for detailed studies on N-linked oligosaccharide profile of therapeutic recombinant monoclonal antibodies. J. Chromatogr. A.

[24] Ma S., Nashabeh W. (1999). Carbohydrate analysis of a chimeric recombinant monoclonal antibody by capillary electrophoresis with laser-induced fluorescence detection. Anal. Chem..

[25] Chen F.-T.A., Evangelista R.A. (1998). Profiling glycoprotien N-linked oligosaccharide by capillary electrophoresis. Electrophoresis.

[26] Guttman A. (1996). High-resolution carbohydrate profiling by capillary gel electrophoresis. Nature.

[27] Gennaro L.A., Salas-Solano O., Ma S. (2006). Capillary electrophoresis-mass spectrometry as a characterization tool for therapeutic proteins. Anal. Biochem..

[28] Campa C., Coslovi A., Flamigni A., Rossi M. (2006). Overview on advances in capillary electrophoresis-mass spectrometry of carbohydrates:A tabulated review. Electrophoresis.

[29] Liu Y., Salas-Solano O., Gennaro L.A. (2009). Investigation of sample preparation artifacts formed during the enzymatic release of N-linked glycans prior to analysis by capillary electrophoresis. Anal. Chem..

[30] Gahoual R., Burr A., Busnel J.M., Kuhn P., Hammann P., Beck A., Francois Y.N., Leize-Wagner E. (2013). Rapid and multi-level characterization of trastuzumab using sheathless capillary electrophoresis-tandem mass spectrometry. mAb.

[31] Rustandi R.R., Anderson C., Hamm M., Beck A. (2013). Application of Capillary Electrophoresis in Glycoprotein Analysis. Glycosylation Engineering of Biopharmaceuticals Methods in Molecular Biology.

[32] Rustandi R.R., Washabaugh M.W., Wang Y. (2008). Applications of CE SDS gel in development of biopharmaceutical antibody-based products. Electrophoresis.

[33] Sheeley D.M., Merrill B.M., Taylor C.E. (1997). Characterization of Monoclonal Antibody Glycosylation: Comparison of Expression Systems and Identification of Terminal α-Linked Galactose. Anal. Biochem..

[34] Beck A., Bussat M.-C., Zorn N., Robillard V., Klinguer-Hamour C., Chenu S., Goetsch L., Corvaia N., Van Dorsselaer A., Haeuw J.-F. (2005). Characterization by liquid chromatography combined with mass spectrometry of monoclonal anti-IFG-1 receptor antibodies produced in CHO and NS0 cells. J. Chromatogr. B.

[35] Ghaderi D., Taylor R.E., Padler-Karavani V., Diaz S., Varki A. (2010). Implications of the presence of *N*-glycolylneuraminic acid in recombinant therapeutic glycoproteins. Nat. Biotechnol..

[36] Galili U. (2004). Immune Response, Accomodation, and Tolerance to Transplantation Carbohydrate Antigens. Transplantation.

[37] Chung C.H., Mirakhur B., Chan E., Le Q.-T., Berln J., Morse M., Murphy B.A., Satinover S.M., Hosen J., Mauro D. (2008). Cetuximab-Induced Anaphylaxis and IgE Specific for Galactose-α-1,3-Galactose. New Engl. J. Med..

[38] Commins S.P., Platts-Mills T.A.E. (2010). Allergenicity of Carbohydrates and Their Role in Anaphylactic Events. Curr. Allergy Asthma Res..

[39] Goetze A.M., Liu Y.D., Zhang Z., Shah B., Lee E., Bondarenko P.V., Flynn G.C. (2011). High-mannose glycans on the Fc region of therapeutic IgG antibodies increase serum clearance in humans. Glycobiology.

[40] Jiang X.-R., Song A., Bergelson S., Arroll T., Parekh B., May K., Chung S., Strouse R., Mire-Sluis A., Schenerman M. (2011). Advances in the assessment and control of the effector functions of therapeutic antibodies. Nat. Rev. Drug. Discov..

